# NMR Based Metabolomic Analysis of Health Promoting Phytochemicals in Lentils

**DOI:** 10.3390/metabo9080168

**Published:** 2019-08-13

**Authors:** Simone Rochfort, Simone Vassiliadis, Pankaj Maharjan, Jason Brand, Joe Panozzo

**Affiliations:** 1 Agriculture Research Victoria, 5 Ring Road, Bundoora, VIC 3083, Australia; 2 School of Applied Systems Biology, La Trobe University, Bundoora, VIC 3083, Australia; 3 Agriculture Research Victoria, 110 Natimuk Road, Horsham, VIC 3400, Australia

**Keywords:** lentil, *Lens culinaris*, trigonelline, kaempferol, catechin, raffinose, ciceritol, hulls, cotyledon

## Abstract

Lentils are a high-protein plant food and a valuable source of human nutrition, particularly in the Indian subcontinent. However, beyond sustenance, there is evidence that the consumption of lentils (and legumes in general) is associated with decreased risk of diseases, such as diabetes and cardiovascular disease. Lentils contain health-promoting phytochemicals, such as trigonelline and various polyphenolics. Fourteen lentil genotypes were grown at three locations to explore the variation in phytochemical composition in hulls and cotyledons. Significant differences were measured between genotypes and environments, with some genotypes more affected by environment than others. However, there was a strong genetic effect which indicated that future breeding programs could breed for lentils that product more of these health-promoting phytochemicals.

## 1. Introduction

Lentil (*Lens culinaris* L.) are consumed worldwide due to their nutrient density and high protein quality [1]. Lentils vary in seed size, seed-coat, cotyledon color and micronutrient properties. Extensive plant breeding has transformed lentils from landraces to regionally adapted commercial cultivars with improved biotic and abiotic tolerance, suitable for mechanical harvesting. There is increasing evidence that lentils contain phytochemicals that provide benefits for human health beyond basic nutrition. The benefits attributed to lentils as well as other pulses include neuronal health; cardiovascular health; and anti-inflammatory, antiviral and antidiabetic activity [2,3,4,5,6,7,8,9,10]. These bioactivities have been largely attributed to the phenolic constituents, in particular the flavanoids, anthocyanins and phenolic acids [11]. In addition, the raffinose series of oligosaccharides, once thought to be solely causing bloating and intestinal distress in monogastrics, are now recognized as important prebiotics for beneficial bacteria that form part of the human gut microbiome [12,13].

The current study uses an NMR metabolomics approach to assess how these lentil metabolites vary between commercially available cultivars and breeding lines when grown at three locations in Victoria, Australia. NMR metabolomic approaches were applied previously to study the geographic and climatic dependencies of green tea metabolites, as well as rice metabolites from rice grown in different parts of China [14,15]. That approach has also been used to study human serum and urine profiles after eating lentils, chickpeas and beans [16], but to our knowledge not to investigate varietal differences of lentils grown under different environmental conditions. 

There have been some studies investigating the chemical constituents of lentils. For example, Tsopmo and Muir investigated the metabolites found in twelve commercial cultivars of lentils grown in Canada detailing the flavanol glycosides, quaternary nitrogen metabolites (trigonelline and hypaphorine) and sugars present in the cultivars, and used this data to cluster them based on metabolic similarity [17]. Zhang et al. studied the phenolic extracts for 20 Canadian lentil cultivars for their composition and bioactivity (inhibition of α-glucosidase and pancreatic lipase) [7]. There are only a few studies on the effects of the environment on the proximal and biochemical properties of lentils. Sehgal et al. investigated the effect of heat stress on eight lentil genotypes by varying the growing time in a single location in India and subjected them to different water stress levels. The authors measured several plant traits, including seed weight and photosynthetic function, as well as proximates and found that under heat-stress, starch was decreased in the seeds while reducing-sugars increased [18]. This trend was confirmed in a more recent study of two lentil genotypes, one heat-tolerant and one susceptible; again the starch was hydrolyzed to increase water soluble carbohydrates in the seed [19]. A study that focused on exploring the influence of environment on seed soluble carbohydrates (the raffinose series of oligosaccharides) in eleven lentil cultivars in ten different environments in Saskatchewan, Canada, found that although there was a statistically significant increase in the concentration of these sugars where rainfall was more limited, the overall effects were not large comparable to the genetic variance, and the broad sense heritability for raffinose family oligosaccharides (RFO) was calculated to be 0.85 [12]. 

NMR metabolomics is a rapid, non-destructive, quantitative method that has potential to be used as part of breeding programs to select for bioactive metabolites in crops. The aim of this study was to assess if NMR based metabolomic approaches could detect differences in bioactive metabolites in the different lentil genotypes grown at three different trial sites in north-western Victoria. 

## 2. Results

**Figure 1:**
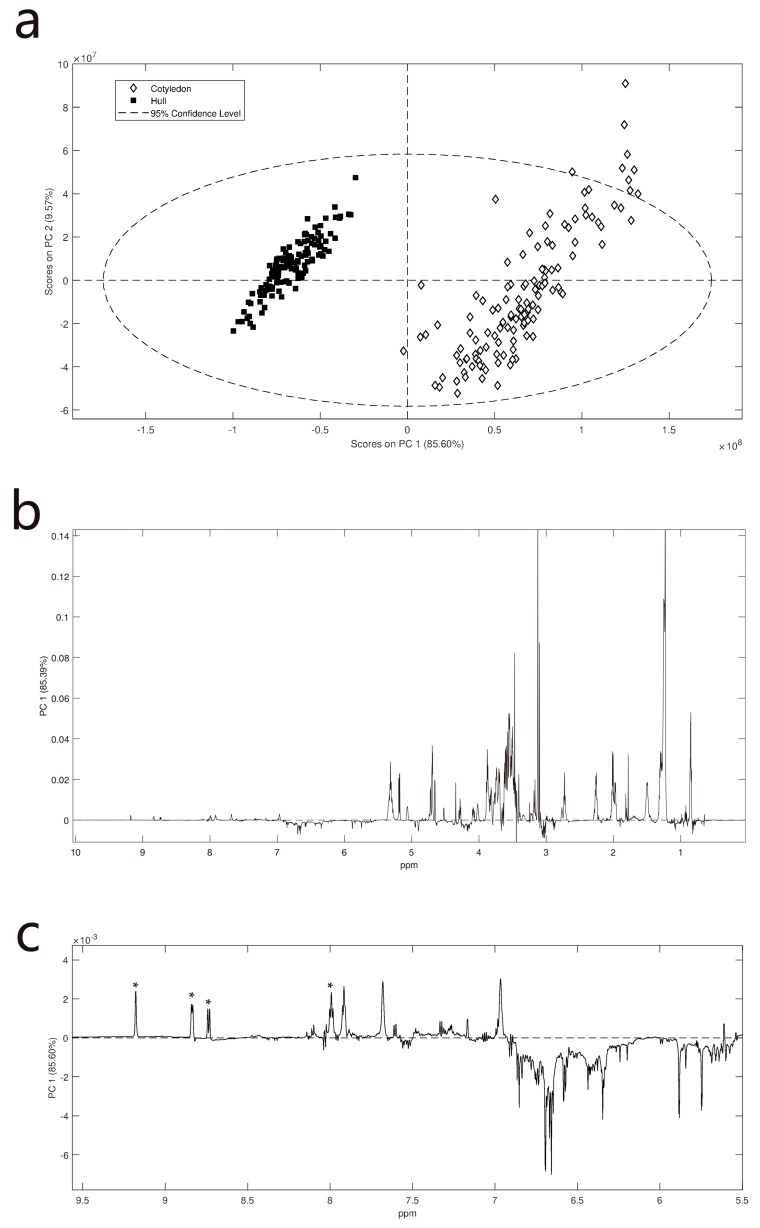
PCA scores and loading plots for ^1^H NMR data of lentil hulls and cotyledon: (**a**) PCA scores plot showing clear separation of hulls and cotyledons regardless of genotype or site; (**b**) loading plot of PC1 variables indicates that the cotyledon has more lipids and carbohydrates; (**c**) expansion of the loading plot focusing on the aromatic region of the spectrum. Downfield signals (above 8.5 ppm) are relatively elevated in cotyledons. Magnetic resonances due to phenolic-containing metabolites (from 5.5 to 8 ppm) are elevated in hulls. Trigonelline resonances indicated by *.

Lentil genotypes consisting of released commercial cultivars and breeding germplasm were grown at three field trial sites in medium and low rainfall zones of northwestern Victoria, Australia over the 2016 growing season. (Meteorological data Supplementary Material Table S1). In 2016 annual and growing season rainfall was significantly higher than average, at approximately 25% greater than the long-term average. In particular, rainfall recorded during September, the key month for reproductive growth, was more than double that of long-term averages. Temperatures throughout the season were consistent with long term averages and no significant frost or heat events were recorded (data not shown). On average, Curyo tends to be warmer (21.3 °C average compared to Rupanyup’s 18.6 °C and Ouyen’s 20.3 °C) and drier (total rainfall from May–December 365.8 mm compared to Rupanyup‘s 509 mm and Ouyen’s 405 mm). The samples were dehulled, and both the seed-coat (hulls) and cotyledons were ground separately and prepared for NMR analysis. The NMR spectra of each lentil fraction was examined by PCA. This unsupervised method describes the variance between the samples. The largest observed difference in NMR spectra was between the cotyledon and hull (Figure 1). This data indicates that the cotyledon contains more lipids, with significant levels of unsaturation, as well as carbohydrates (Supplementary Material Figure S1). Comparison of data for sugar standards with that of a cotyledon (Supplementary Material Figure S2) shows that ciceritol, sucrose and the raffinose sugars were the major water-soluble carbohydrates present. An expansion of the downfield region of the loadings-plots, showed that the hulls, in general, contain more phenolics. In the phenolic region, the presence of catechins dominates the spectra (Supplementary Material Figure S3). The notable exception to this was the resonances associated with trigonelline which was elevated in the cotyledons (Figure 1c, Supplementary Material Figure S4). Since tissue type was the predominant distinguishing variable for the NMR spectra, the spectra were separated out on this basis so that effects of site and variety on each tissue could be investigated.

### 2.1. Lentil Cotyledon

**Figure 2:**
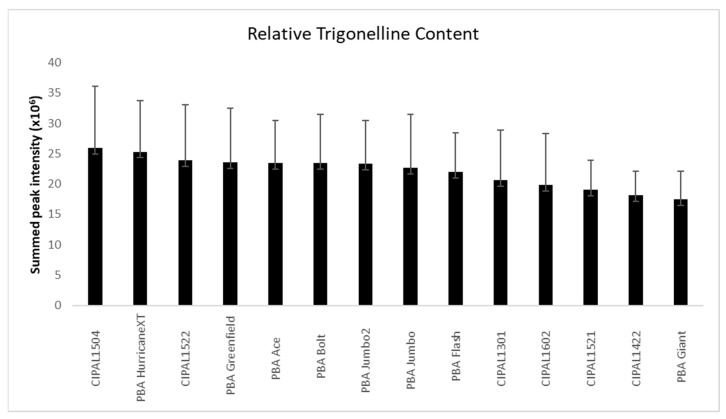
Relative trigonelline content in the lentil genotypes with standard deviation (error bars).

Magnetic resonances for the aromatic alkaloid trigonelline suggested that this molecule was quite abundant in the cotyledons of the lentil genotypes. The identity of trigonelline was confirmed by comparison of NMR data (1 and 2D) to the data in the Human Metabolome Data Base (HMDB) where the resonances matched well, given the different solvents used [20]. Trigonelline has previously been reported in pulses, including lentils, with concentrations of (250 to 1211 μg/g) [21,22]. Trigonelline been found in human urine and has been proposed as a biomarker of pulse consumption [16]. Trigonelline is also found in fenugreek and coffee. Trigonelline is of interest, as several studies have shown that it may be beneficial to treat or prevent diabetes and the symptoms of diabetes, hearing loss, and nonalcoholic fatty liver disease [23,24,25,26,27,28,29]. For this reason, the levels of trigonelline were investigated further in our population. To do this, the region in the NMR spectrum from 8.50 to 9.45 ppm was extracted from the whole spectra and summed for each sample. That spectral region has few resonances, and those present are due to trigonelline alone. The result was, therefore, an approximation of relative trigonelline content in each sample. There was considerable variation in trigonelline (up to 2.2-fold between the 125 cotyledon samples) including variation between genotypes and sites. Despite this, if we examine the average trigonelline content based on lentil variety/line, there are some interesting trends, though the differences are not statistically significant by ANOVA (Figure 2). For example, the genotype CIPAL1504 produces, on average, 32% more trigonelline than PBA Giant (*p* < 0.05). The commercial variety, PBA HurricaneXT, also produces high levels of trigonelline, though not significantly different in trigonelline content to CIPAL1540. 

If the same calculations are made based on growing location rather than genotype, then there is no significant difference between the sites, though there is a trend suggesting trigonelline at the Curyo site is somewhat elevated.

**Figure 3:**
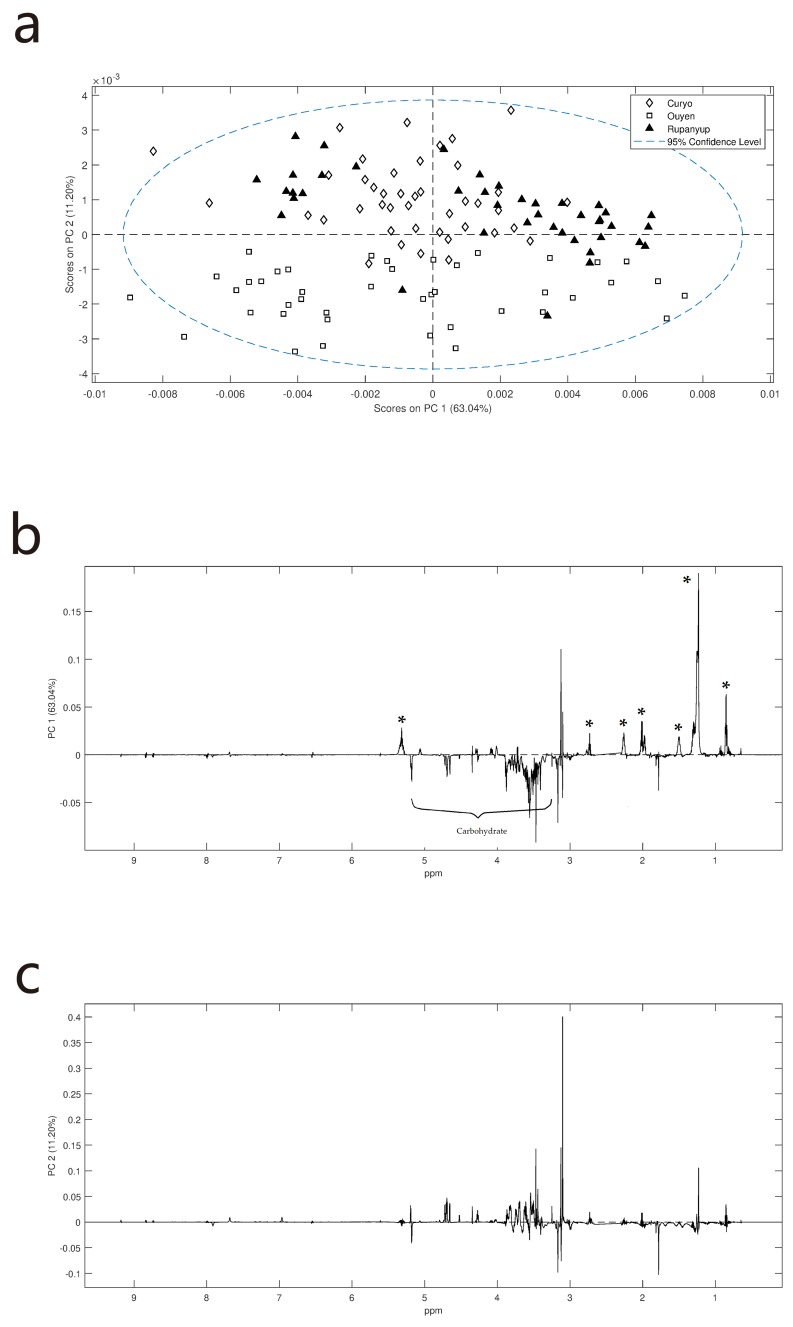
PCA scores and loading plots for ^1^H NMR data of lentil cotyledon: (**a**) PCA scores plot showing some separation based on site in PC2; (**b**) loadings plot of PC1 variables suggest that major variation is in lipids (* in positive PC1) and carbohydrate (resonances between 3.2–5.2 ppm for negative PC1) levels; (**c**) loadings plot of PC2 variables suggest that major variation is in trigonelline and carbohydrate levels.

PCA (principal components analysis) was explored to determine if any of the other metabolites within the cotyledon that were affected by the growing location. (Figure 3). The major separation in PC1 (principal component 1) (Figure 3b) is due to differences in lipids and carbohydrates, with samples on the positive PC1 axis having relatively higher concentrations of lipids and less carbohydrates. To some extent this is influenced by genotype; for example, at Ouyen the CIPAL 1522 lentils have more lipid and less carbohydrates than the PBA Jumbo2 (Supplementary Material Figure S5). There is also some separation by location on PC2 (Figure 3a). The loadings plot (Figure 3c) suggests that lentils grown at Curyo and Rupanyup have elevated levels of trigonelline and carbohydrates.

**Figure 4:**
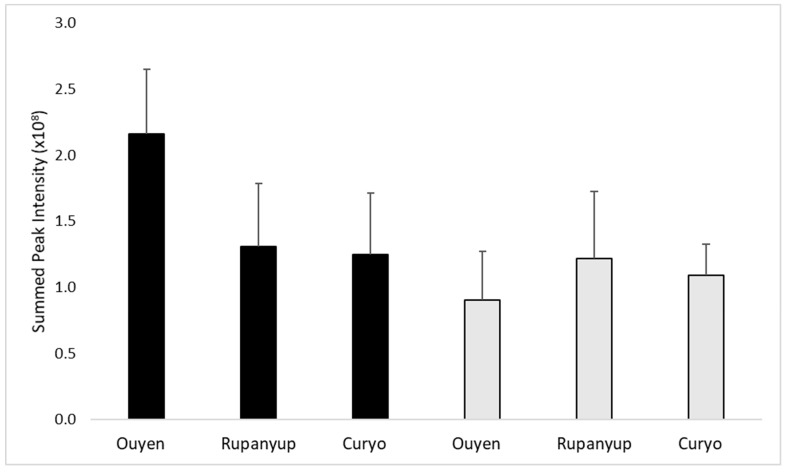
Relative content and standard deviation of carbohydrates in lentil cotyledons across replicates and sites of carbohydrates in the lentil cotyledons for PBA Jumbo2 (black bars) and CIPAL1602 (grey bars). (O = Ouyen, R = Rupanyup, C = Curyo). The PBA Jumbo2 samples from Ouyen have more carbohydrate content compared to Rupanyup (*p* = 0.02) and Curyo (*p* = 0.08). There is no significant difference between content for CIPAL1602 across the three sites (*p* > 0.2).

The main carbohydrates present in lentils are the raffinose series of oligosaccharides and sucrose [12]. Sucrose is a disaccharide of glucose and fructose. Raffinose is a trisaccharide composed of galactose, glucose, and fructose. Stachyose is a tetrasaccharide with galactose, galactose, glucose and fructose in linkage. Verbascose is a pentasaccharide composed of galactose, galactose, galactose, glucose and fructose. As well as these carbohydrates, ciceritol, a galactosyl cyclitol consisting of galactose, galactose and 4-*O*-methyl-inositol, is also abundant in lentils [30]. Sucrose is a common subunit of the raffinose series and the galactose-galactose subunit is also common to ciceritol; therefore, there was overlap in the ^1^H NMR spectra due to these carbohydrates (Supplementary Material Figure S2). This made quantification from NMR problematic. However, the raffinose series, ciceritol and sucrose, had resonances between 5.14 and 5.22 ppm that are not overlapping with other resonances. PCA of this data revealed a complex pattern with no overall obvious clustering (data not shown). One-way ANOVA showed there were no significant differences between the varieties when data from all sites were considered. However, close examination of the data showed that there was a complex interaction between environment and genotype. Comparison of all samples showed there was a 4.2 fold difference between the highest and lowest levels of carbohydrates. However, comparison of these two extreme genotypes, PBA Jumbo2 and CIPAL1602, showed that this was also strongly associated with location (Figure 4). Although there is no significant difference between the CIPAL1602 carbohydrate levels, a greater number of replicates may decrease the sample variation. 

### 2.2. Lentil Hull

**Figure 5:**
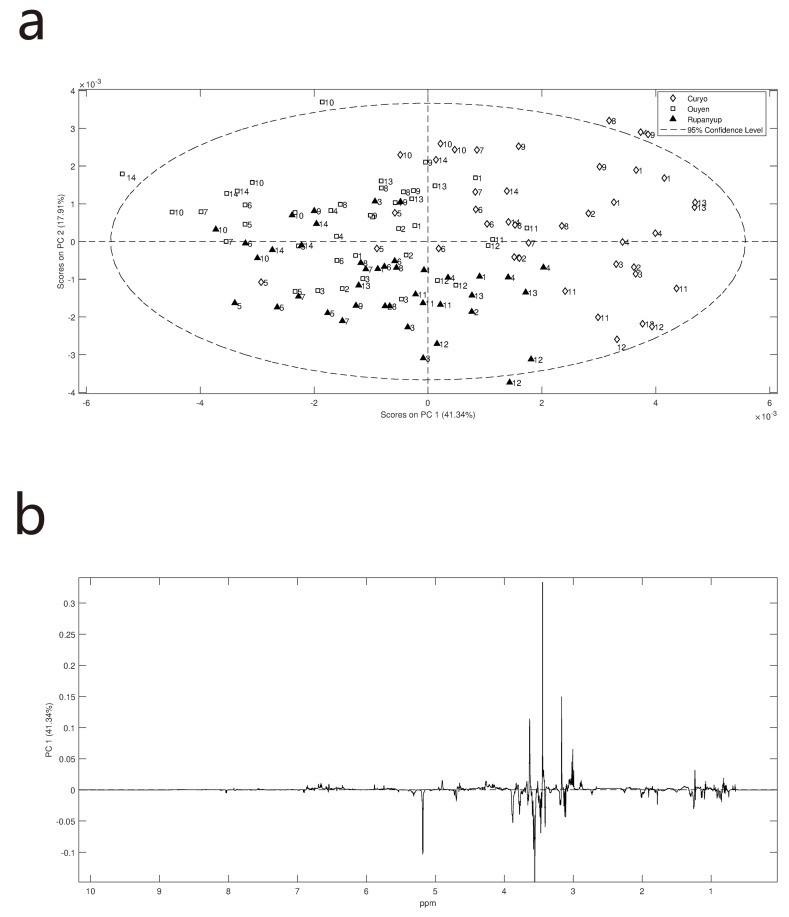
PCA scores and loading plots for ^1^H NMR data of lentil hulls: (**a**) PCA scores plot showing some separation based on site and genotype in PC1 (1 = CIPAL1301, 2 = CIPAL1422, 3 = CIPAL1504, 4 = CIPAL1521, 5 = CIPAL1522, 6 = CIPAL1602, 7 = PBA Ace, 8 = PBA Bolt, 9 = PBA Flash, 10 = PBA Giant, 11 = PBA Greenfield, 12 = PBA HurricaneXT, 13 = PBA Jumbo, 14 = PBA Jumbo2); (**b**) Loadings plot of PC1 variables suggest that major variation is in carbohydrate levels (3.2–5.2 ppm) with phenolics (6.2–7.3 ppm) and amino acids (branched chain amino acids: Leucine, isoleucine, valine, all 0.8–1.2 ppm) also influencing the loadings.

The hulls contained less carbohydrate compared to the cotyledons, and spectra from the hulls were rich in resonances due to phenolics and aromatic metabolites. PCA of the hull data suggested that location was a strong influencer for Curyo, where most of the samples partitioned in positive PC1 quadrants (Figure 5a), indicating a relative increase in phenolic resonances and a decrease in carbohydrate resonance (Figure 5a), indicating a relative increase in phenolic resonances and a decrease in carbohydrates (Figure 5b). Interestingly, there are some genotypes for which this is not the case. For example, the CIPAL1522 spectra tended to cluster together, suggesting that this genotype is less susceptible to environmental change than some of the others. 

**Figure 6:**
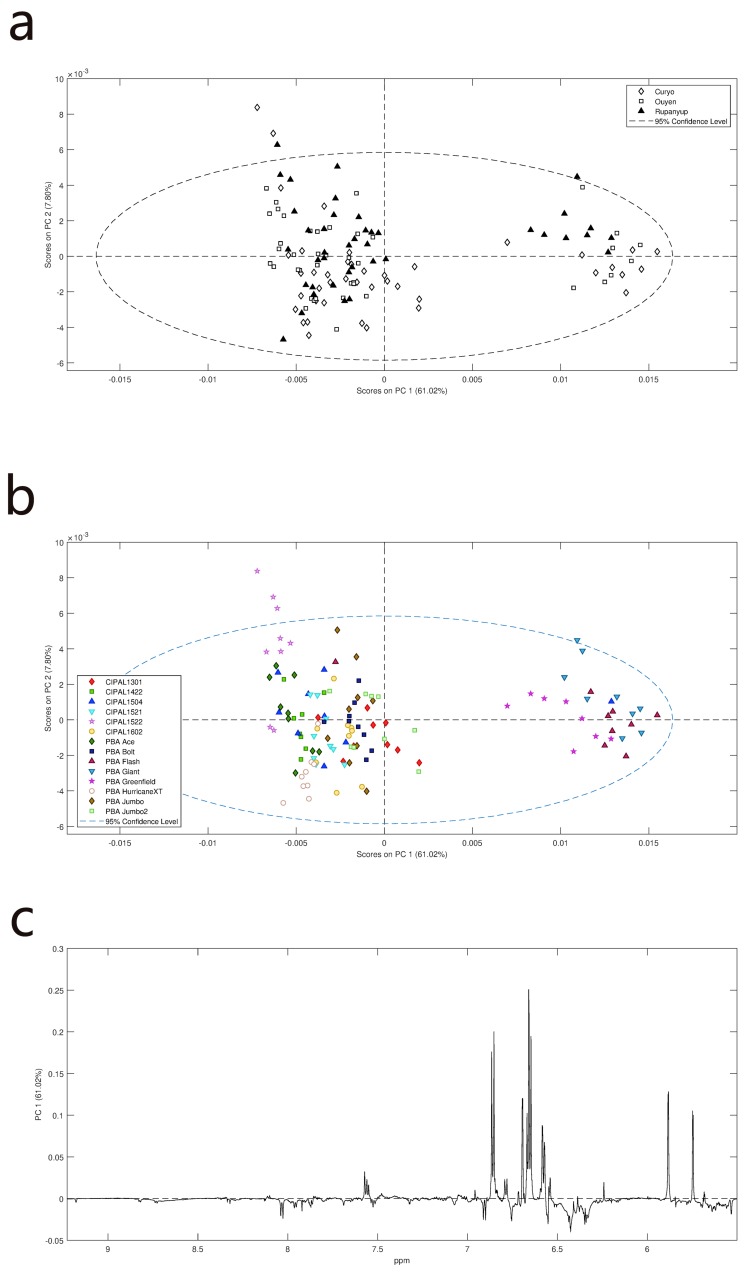
PCA scores and loadings plots for ^1^H NMR data of lentil hulls: (**a**) PCA scores plot colored according to site; (**b**) PCA scores plot colored according to genotype; (**c**) loadings plot of PC1 variables suggest that major variation is in the levels of catechin/epicatechin derivatives and kaempferol.

In order to explore the effect of genotype and location on the phenolic constituents, the upfield portions of the spectra (resonances below 5.5 ppm) were removed. PCA of that data reveals that the major separation was due to variety rather than location with levels of catechin/epicatechin derivatives being the major influence on separation (Figure 6). However, aromatic resonances due to the kaempferol a\were also influencing the loadings, in the opposite direction to the catechin/epicatechin signals.

It appears that the concentrations of catechin/epicatechin derivatives are higher in green lentils PBA Giant, and PBA Greenfield, and in the medium-sized red lentil, PBA Flash, compared to the small-seed-size lentils. There was also an effect of location that was seen when only those three genotypes were analyzed by PCA, which suggests those phenolics were elevated, while others were observed to decrease at the Curyo site compared to Ouyen or Rupanyup (Supplementary Material Figure S6). 

The core catechin/epicatchin subunit was identified by comparison of 1D and 2D NMR data (Supplementary Material Figure S7) for a hull sample with literature and with a standard [20,31]. It appears that in PBA—for Giant, Greenfield and Flash, the catechin/epicatechin is derivatized, most likely through glycosylation. In the 2D-NMR data a long-range C–H correlation can be seen from the flavanol–3H (4.14, 73.0 ppm) to a resonance at 101.3 ppm. The heteronuclear single quantum coherence (HSQC) spectral data shows that this carbon is attached to a proton that resonates at 4.17 ppm, indicative of an anomeric proton, suggesting that the catechin/epicatechin is glycosylated. The 1D NMR spectrum of the crude extract is too complex to enable the nature of the glycosylation to be determined, since the sugar region between 3–4 ppm is very congested (Supplementary Material Figure S1). It is possible that there are different sugars attached to the catechin/epicatechin, but this would be best resolved by a liquid chromatography mass spectrometry (LCMS) based approach or isolation and structure elucidation of the metabolites. The resonances of the kaempferol moiety were assigned by comparison to literature data for a kaempferol glycoside, the data for which was also obtained in DMSO [32]. It is likely that the kaempferol is also present as a glycoside, although the signals in the 2D NMR were not strong enough to confirm this. However, several kaempferol glycosides have been previously identified in lentils and other pulses [7]. Again, LCMS analysis would be useful to define the number of kaempferol glycosylated species and would provide additional information about the number of sugars present in each species.

**Figure 7:**
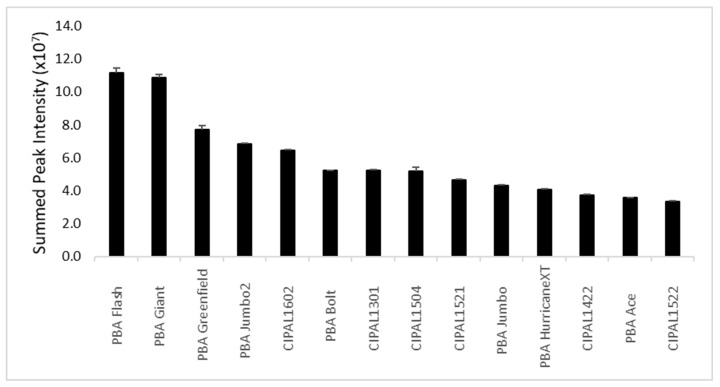
Mean and standard deviation of catechin/epicatechin content in lentil hulls for all samples across the three growing locations.

Since the health benefits of catechins are well known and include neuronal health, cardiovascular health, anti-inflammation, and antiviral and antidiabetic activities [2,3,4,5,6,7,8], it was decided to extract relative catechin/epicatechin levels in the data set by selecting the region between 5.875 and 5.893 ppm, the part of spectra where the resonance due to catechin has the least overlap with any other resonances. As indicated by the PCA data, PBA Flash, PBA Giant and PBA Greenfield are the three commercially-released cultivars that contain the most catechin/epicatechin. PBA Flash contains three times more compared to CIPAL1522 (Figure 7).

If the same data is analyzed for each site, then the Ouyen site produces the highest levels of catechins; however, the difference is not significant due to the large variation due to genotype differences (Supplementary Material Figure S8a). However, if only PBA Flash, PBA Giant and PBA Greenfield are considered, then the lentils grown at Ouyen contain significantly more catechin (*p* < 0.05) (Supplementary Material Figure S8b).

**Figure 8:**
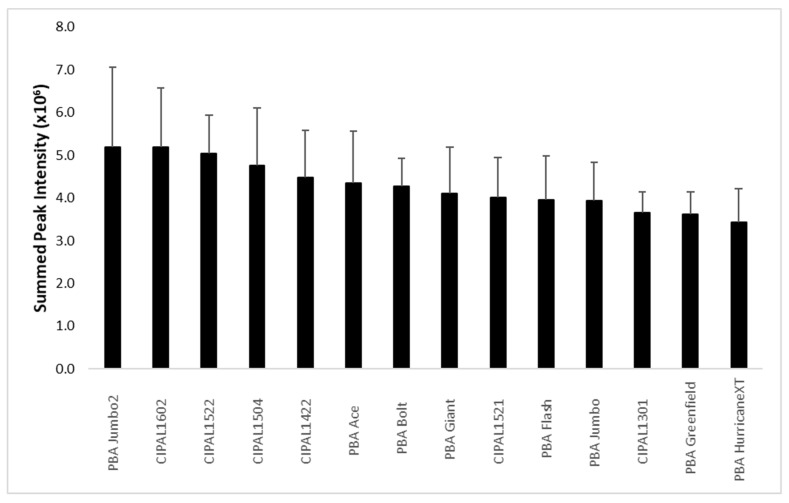
Relative content and standard deviation across reps and sites of kaempferol derivatives in the lentil hulls.

Flavanols are also known to possess similar health properties. The loading plot suggests that the major flavanol is kaempferol, and that it accumulates in an opposite manner to the catechins (Figure 6c). To explore this, the kaempferol resonance that was least overlapping with other metabolites was extracted (8.00 to 8.06 ppm) and the data summed up to give a relative concentration of kaempferol in each variety (Figure 8). This data shows that there is less variation in kaempferol compared to catechin; catechin levels vary 3-fold while kaempferol varies 1.5-fold across the varieties. However, the cultivar with the highest kaempferol levels, PBA Jumbo2, contains significantly more than the lowest, PBA HurricaneXT (52%, *p* < 0.05), but 60% less catechin than PBA Flash (the cultivar with the highest amount of catechin). Overall the cultivars contain approximately 13 times more catechin than kaempferol. Interestingly, this is in contrast to a previous Canadian report, where red lentils were shown to have similar levels of these metabolites, and green lentils had more kaempferol than catechins [7]. However, our analysis shows that there is also an environmental effect on the levels of kaempferol with Ouyen collectively having the highest levels and Curyo the lowest (Supplementary Material Figure S9).

## 3. Discussion

This study has shown that bioactive constituents in lentils can be affected by both genetics and the environment to different extents depending on the metabolite measured. There was considerable variation for some metabolites, even at one site. This suggest that future studies should include more replicates for assessment of these traits. One of the bioactives present in cotyledons but not (or at very low levels) in hulls was trigonelline. There have been only a few studies on the levels of trigonelline in lentil cotyledons or edible seedlings [22,33,34]. The study by Rozan et al. compared *L. culinaris* seeds and seedlings with other *Lens* species. They showed that there was diversity in the accumulation of trigonelline in the *Lens* species (0–18 mg/g), with the cultivated genotype containing 12 mg/g [22]. The same authors later compared lentils with other legume species, and reported 0.17 mg/g trigonelline in lentil cotyledons from commercially available sprouted seedlings [33]. De Zwart et al. measured the levels of trigonelline and other non-proteinaceous amino acids in a large range of common foods and found trigonelline at 0.25 mg/g [21]. Trigonelline is believed to have a role in protection against environmental stress, including salt tolerance [35] and in preventing water loss [33]. In our study we did not see a significant variation in trigonelline levels according to location, although it might have been expected from the literature that the metabolite would be elevated in the warmer, dryer site Ouyen. However, we did see considerable variation between genotypes with a variation of 2.2-fold between the highest and lowest levels of trigonelline. This suggests that there is a significant genetic component to the levels of trigonelline in lentils, and there is; therefore, there is the opportunity to breed for increased levels of this health promoting metabolite.

It has been previously shown that accumulation of the raffinose family of oligosaccharides is highly heritable in lentils, with heritability estimates of 0.85 [12]. However, under heat stress conditions during seed filling, the water-soluble sugars can increase at the expense of starch and protein [19]. Tahir et al. studied eleven lentil genotypes in eight environments in Canada showing that relatively dry environments led to elevated levels of RFO (raffinose, stachyose and verbascose) but also noted the high genetic component of RFO concentration [12]. Our study produced similar conclusions, observing considerable variation in the accumulation of carbohydrates in our genotypes that was also influenced by site. Overall, the warmer, dryer environment in Ouyen produced higher levels of water-soluble carbohydrates (WSC) in many of the genotypes. However, there were also genotypes that produced less WSC, for example CIPAL1602, which had similarly low levels of WSC across the three sites. This may be an indication that CIPAL1602 is less responsive and perhaps more tolerant of heat or water stress, although more field trials would be needed to confirm this. It would also be interesting to determine the quantity of individual sugars in these samples. Tahir et al. found that stachyose, raffinose and verbascose content of lentil genotypes ranged from 22.0–25.5, 19.5–22.2 and 11.5–13.3 mg/g respectively, but did not measure ciceritol [12]. Other reports confirm the variation in WSC content and in individual carbohydrates, with ciceritol, raffinose and stachyose being present in in quantities between 20 and 50 mg/g, with verbascose significantly lower (3.9–7.9 mg/g) [30].

The phenolic content of lentils is thought to be at least partially responsible for their beneficial health effects [10,11]. The hulls contain relatively more of the phenolic compounds compared to cotyledon. Previous studies on the phenolics in lentil have identified that catechins, flavanols and proanthocyanidins contribute to the total phenolics of lentils [7,17,36,37,38,39]. In the present study we saw that genotype had a major influence on phenolic content, but that can be influenced by the environment. PBA Flash (medium red lentil with tan seed coat), PBA Giant (large green lentil with a light green seed coat) and PVA Greenfield (medium green lentil with a light green seed coat) were the three cultivars that contained the most catechin/epicatechin derivatives, and their levels were highest in the samples grown at Ouyen. The other varieties, all red lentils with grey or tan seed coat had relatively less of the catechins and more of the kaempferol derivatives. The PBS Jumbo genotype (medium red lentil with a grey seed coat) had the highest levels of kaempferols. The relationship between seed coat color and flavonoid content was recently investigated by Marili et al. [37]. They identified that kaempferol digylcoside (tentatively identified as kaempferol dirutinoside) was the major flavanol but also identified several other kaempferol derivatives, as well as quercetin, myricetin and luteolin glycosides. They found greater concentrations of flavanols, flavan-3-ols and proanthocyanidins in lentils with green and gray compared to tan and brown seed coats. Like the current study they found that catechin 3-*O*-β-d-glucoside was highest in green and grey seed coats. The lentils in the study by Mirali et al. were grown at two different locations in Canada and noted that there was an effect of location and that this effect was greater on some metabolites (e.g., proanthocyanidins and some catechins) compared to others (e.g., kaempferol dirutinoside) which broadly correlates with our observations. 

## 4. Materials and Methods 

### 4.1. Plant Material

A total of 14 lentil genotypes were sourced from Agriculture Victoria’s germplasm enhancement program representing a range in market grades defined by seed-shape characteristics, seed-coat color and cotyledon color phenotypes (Supplementary Material Table S2). The genotypes were grown in three field trials exhibiting diverse soil characteristics and environmental conditions defined by temperature and rainfall (Supplementary Material Table S1). The experimental design was a randomized complete block with three replications for each genotype.

Following harvest, grain was aspirated to remove only plant material retaining all seeds. The seeds were dehulled and the seed-coat (hulls) and cotyledons ground to a fine powder with a particle size of less than 0.5 mm.

### 4.2. Chemicals

All chemicals used throughout the study were of the highest quality. Methanol (>99.9% pure) was purchased from Fisher Chemical (Fair Lawn, NJ, USA). Dimethyl Sulfoxide-D_6_ (DMSO, >99.5% pure) was purchased from Cambridge Isotope Laboratories, Inc. (Tewksbury, MA, USA). For confirmation of sugars, d-mannitol (>98% pure), sucrose (>99.5% pure), d-(+)-raffinose pentahydrate (>98% pure), and d-(+)-trehalose dihydrate (>99.0% pure) were purchased from Sigma-Aldrich (St Louis, MO, USA. d-glucose (>99% pure) and inositol (>98%) were purchased from Cambridge Isotope Laboratories, Inc. and d-(-)-fructose (>98% pure) was purchased from BDH Analytical (Poole, UK). Ciceritol was isolated in-house (Briefly, a water extract of chickpeas was purified by reverse phase C18 HPLC, to afford ciceritol). Two phenolic compounds, (+)-catechin hydrate (98%) and (-)-epicatechin, were purchased from Sigma-Aldrich.

### 4.3. Sample Preparation for NMR

The ground hull and cotyledon samples (50.01 ± 0.01 mg dried weight) were extracted two times with 1 mL of 80% methanol: 20% milli-Q water (v:v) followed by vortexing (Ratek multi tube vortex mixer, MTV1), sonication (SoniClean, 250TD), and centrifugation (16,100 *g*, 21 °C). The extracts were combined and dried under vacuum at 21 °C overnight (SpeedVac Concentrator, Thermo Fisher Scientific, Savant SPD 2010). The dried extracts were reconstituted in DMSO-d_6_ (600 μL) and 550 μL aliquots were transferred to 5 mm NMR tubes for analysis.

### 4.4. NMR Spectroscopy

Proton spectra were obtained on a Bruker 700 MHz instrument (Bruker Biospin, Germany) equipped with a cryoprobe. A Bruker noesypr1d pulse sequence was used over −15 to 15 ppm spectral range with 160 scans collected after a dummy scan; total acquisition time was 1.14 s. A line broadening of 0.3 Hz was applied to all spectra prior to Fourier transformation. Spectra were manually phased, and baseline corrected in Topspin 3.5 (Bruker Biospin, Germany). Samples were referenced to residual DMSO (2.54 ppm). 2D gradient NMR spectra were acquired using the Bruker pulse sequences cosygpqf, hsqcedetgpsisp2.2 and hmbcgpl2ndqf with 160, 192 and 256 scans respectively. Pulsecal was used to determine the 90-degree pulse for each standard and for the lentil hull and cotyledon samples. 

### 4.5. Statistical Analysis

Data, a total of 12719 bins, were imported into Matlab (R2018a, Mathworks) as previously described [40]. Statistical analysis including principal component analysis (PCA), was performed using PLSToolbox (Ver 8.6.1, Eigenvector Research). The data were reduced to regions of interest and pre-processed prior to model building. Pre-processing included baseline adjustment (automatic weighted least squares, order −2), normalization (total spectral area normalized to 1) and mean centering.

Specific bins were summed to estimate individual metabolite levels: For trigonelline the spectral region from 8.50 to 9.45 ppm (1037 bins) was extracted and summed; for carbohydrates the region from 5.14 and 5.22 ppm (89 bins) was used; for catechin and epicatechin the region from 5.875 and 5.893 ppm (19 bins) was use; and for kaempferol, the region 8.004 to 8.060 ppm (62 bins) was used. These areas were used to calculate mean, standard deviation and to plot bar charts of metabolite levels. 

One-way ANOVA with Tukey’s HSD and multiple comparison tests were carried out in MatLab for metabolite variables to assess differences between genotypes or location. 

In specific cases, e.g., to compare highest and lowest samples, a student’s *t*-test (unpaired, two-tailed distribution) was also used to calculate *p* values for metabolites for two way comparisons.

## 5. Conclusions

Rapid profiling by ^1^H NMR metabolomics has revealed both genotypic and environmental effects in the accumulation of metabolites that potentially contribute to improved metabolic health. The cotyledon was found to be richer in water soluble carbohydrates, especially ciceritol. Although the raffinose series of oligosaccharides are known to impact on digestion and cause flatulence, they may also be important as prebiotics. The health effects of ciceritol have not been well explored. The hulls also contained significant amounts of trigonelline, a metabolite which has demonstrated both in vitro and associated in vivo benefits for animal health. The hulls are a rich source of polyphenolics, the levels of which are largely genetically determined, although the composition and concentration are influenced by abiotic effects. Our data indicates that the levels of these metabolites may be beneficially exploited through genetics and optimized agronomy practices. 

## Supplementary Materials

The following are available online at https://www.mdpi.com/2218-1989/9/8/168/s1: Table S1: Seed characteristics of 14 lentil genotypes; Table S2: Monthly total rainfall and monthly mean maximum temperature during lentil growing season in 2016 at three trial sites. Figure S1: ^1^H NMR spectra of hull and cotyledon for lentil line CIPAL1422 grown at Ouyen showing resonances associated with the major metabolite classes present. Figure S2: ^1^H NMR spectra of a lentil and sugar standards. Figure S3: ^1^H NMR spectra of a lentil hull and catechin/epicatechin standards. Figure S4: ^1^H NMR spectra of hull and cotyledon for lentil line CIPAL1422 grown at Ouyen showing downfield resonances associated with the major aromatic metabolite classes present. Figure S5: PCA scores plot for cotyledon data colored by variety with site indicated by letter. Figure S6: PCA scores and loadings plot for phenolic region hull data for PBA Giant, PBA Flash and PBA Greenfield demonstrating variance according to site in PC2. Figure S7: 2D NMR data for a hull sample. Figure S8: Relative catechin content and standard deviation in the lentil hulls by site. Figure S9: Relative kaempferol content and standard deviation in the lentil hulls by site. Table S3: (a–i) NMR data for standards, assigned by 1H NMR (noesypr1d), 2D NMR (cosygpqf, hsqcedetgsisp2.2 and hmbcgpl2ndqf) and references where noted.
