# Supplementary material for: NMR Based Metabolomic Analysis of Health Promoting Phytochemicals in Lentils
Source: Metabolites. 2019 Aug 13;9(8):168. doi: 10.3390/metabo9080168 (PMC6724105; doi:10.3390/metabo9080168)
Supplement: Supplementary file 1 [file metabolites-09-00168-s001.pdf]

Supplementary Data

NMR based metabolomic analysis of genetic and regionality on health promoting phytochemicals in lentil

Simone Rochfort [1,2]*, Simone Vassiliadis [1], Pankaj Maharjan [3] Jason Brand [3] and Joe Panozzo [3]

[1] Agriculture Research Victoria, 5 Ring Road, La Trobe University, Bundoora VIC 3083, Australia;
[2] School of Applied Systems Biology, La Trobe University, Bundoora VIC 3083, Australia;
[3] Agriculture Research Victoria, 110 Natimuk Road, Horsham VIC 3400, Australia;

* Correspondence: simone.rochfort@ecodev.vic.gov.au;

Supplementary Table 1. Seed characteristics of 14 lentil genotypes.

Cultivar/genotype	Market grade	Seed phenotype	Seed coat colour phenotype	Cotyledon colour phenotype	Intended end use
PBA Giant	Large green	Plump lens	Light green	Yellow-green	Not split
PBA Greenfield	Medium green	Lens	Light green	Yellow-green	Split
PBA HurricaneXT	Small red	Plump-round	Grey	Red-orange	Split
PBA Jumbo2	Large red	Lens	Grey	Red-orange	Split
CIPAL1602	Medium red	Round lens	Grey	Red-orange	Split
CIPAL1301	Medium red	Lens	Grey	Red-orange	Split
PBA Flash	Medium red	Lens	Tan	Red-orange	Split
CIPAL1522	Medium red	Lens	Grey	Red-orange	Split
PBA Bolt	Medium red	Lens	Grey	Red-orange	Split
PBA Ace	Medium red	Lens	Grey	Red-orange	Split
PBA Jumbo	Large red	Lens	Grey	Red-orange	Split
CIPAL1504	Medium red	Lens	Grey	Red-orange	Split
CIPAL1422	Medium red	Round lens	Grey	Red-orange	Split
CIPAL1521	Small red	Round lens	Grey	Red-orange	Split

Supplementary Table 2a. Monthly total rainfall and monthly mean maximum temperature during lentil growing season in 2016 at three trial sites.

	Trial site	May	Jun	Jul	Aug	Sep	Oct	Nov	Dec
**Monthly total rainfall** - mm	Curyo	53.5	41.5	50.5	39.5	117.5	75.5	21.0	6.0
	Ouyen	48.9	39.4	31.6	35.2	131.9	47.3	26.2	5.3
	Rupanyup	80.0	52.4	72.0	49.2	143.2	58.0	31.6	22.6
**Monthly mean Max. Temp.- °C**	Curyo	19.5	14.7	14.6	16.5	17.7	21.3	26.9	30.9
	Ouyen	20.3	15.4	15.5	17.6	18.7	22.7	27.7	32.3
	Rupanyup	18.0	13.8	13.2	15.2	16.1	18.4	24.7	29.0

Meteorological data were obtained from The Bureau of Meteorology

Supplementary Table 2b. Trial site location, indicative soil type and annual and growing season rainfall (April – October; GSR) for 2016 in comparison to long term (at three trial sites where lentils were grown.

**Site**	**GPS**	**Soil Type**	**Rainfall (mm)**			
			**2016**[1]		**Long Term**[2]	
			**Annual**	**GSR**	**Annual**	**GSR**
**Ouyen**	35.07°S; 142.32°E	Loamy sand	410	335	331	213
**Curyo**	35.46°S; 142.46°E	Sandy Loam	471	356	374	266
**Rupanyup**	36.32°S; 142.38°E	Black cracking clay	511	384	413	284

1. Data from on-site weather stations at Curyo and Rupanyup and from Ouyen Bureau of Meteorology station (BOM).
2. Longterm data for Ouyen: Ouyen BOM station (1911-2016); Curyo: Beulah BOM station 1898-2016; Rupanyup: Longrenong BOM station 1860-2016.

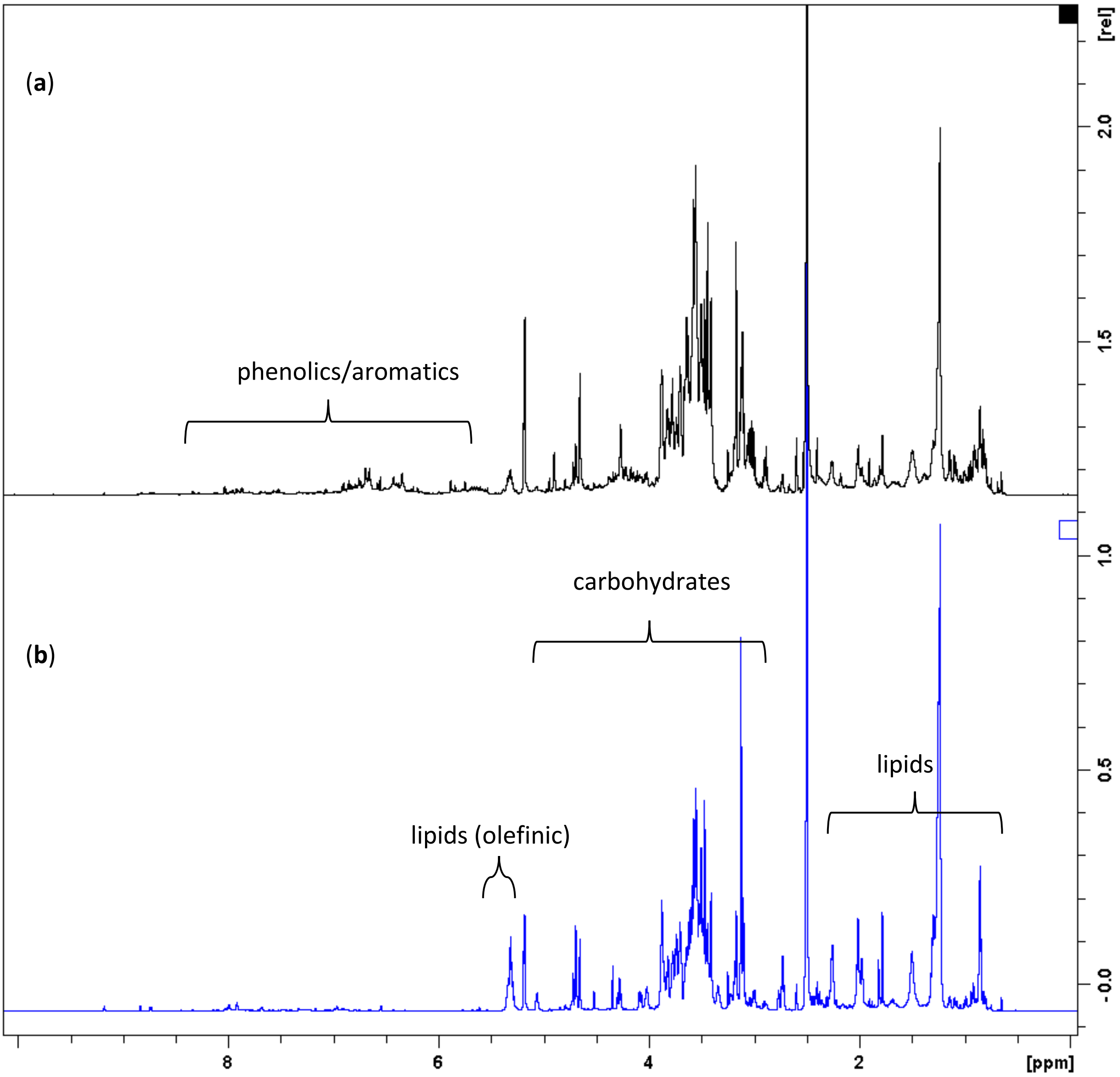

Supplementary Figure 1. ^{1}H NMR spectra of representative hull and cotyledons (lentil line CIPAL1422 grown at Ouyen) showing resonances associated with the major metabolite classes present: **(a)** spectrum of the lentil hull showing more phenolic resonances; and **(b)** spectrum of the lentil cotyledon showing predominance of lipids and carbohydrates.

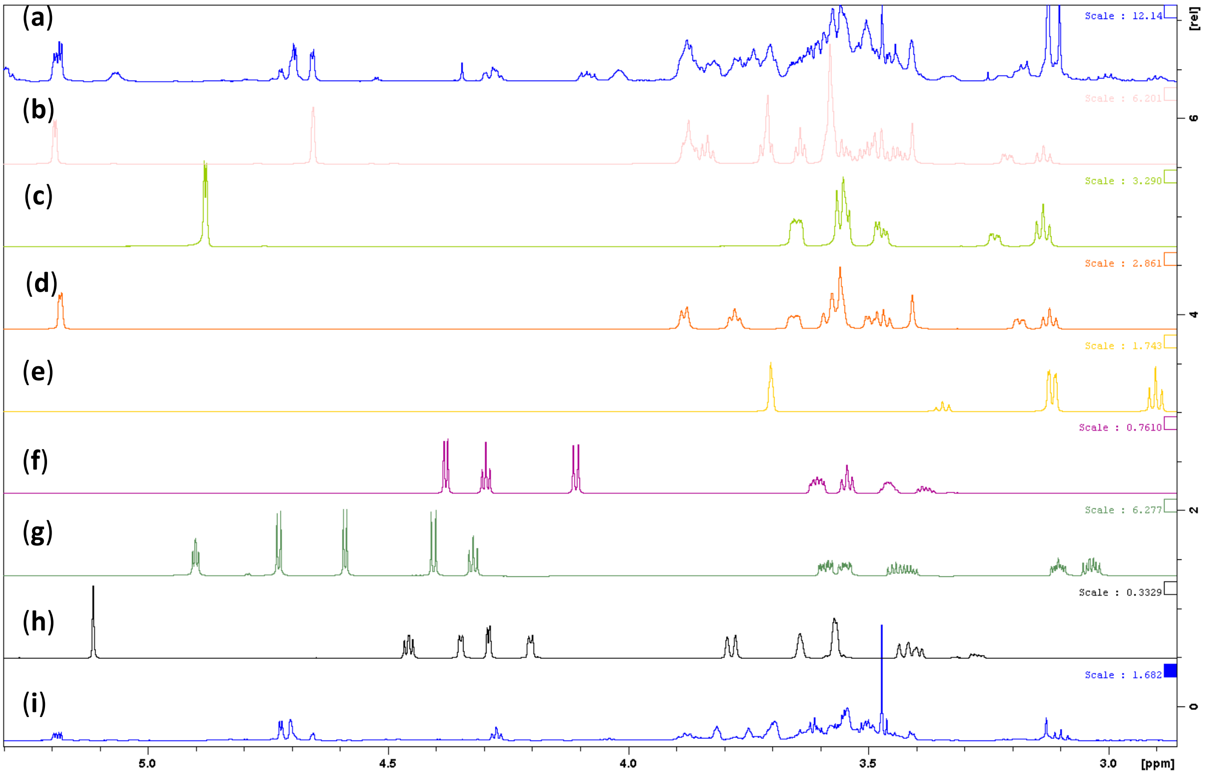

Supplementary Figure 2. ^{1}H NMR spectra of a representative lentil sample compared to sugar standards: **(a)** cotyledon from PBA Flash (Ouyen), **(b)** raffinose, **(c)** trehalose, **(d)** sucrose, **(e)** inositol, **(f)** mannitol, **(g)** α-glucose, **(h)** fructose and **(i)** ciceritol (approx. 50% pure).

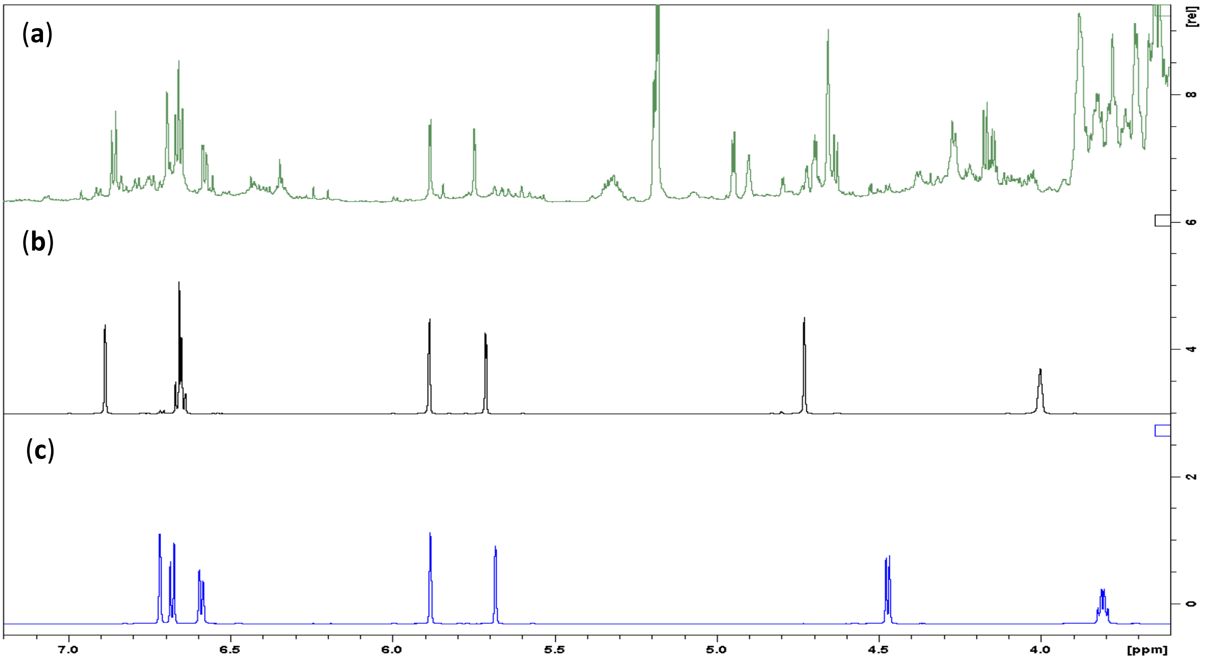

Supplementary Figure 3. ^{1}H NMR spectra of a representative lentil hull and catechin/epicatechin standards: **(a)** hull from PBA Flash (Ouyen), **(b)** epicatechin and **(c)** catechin.

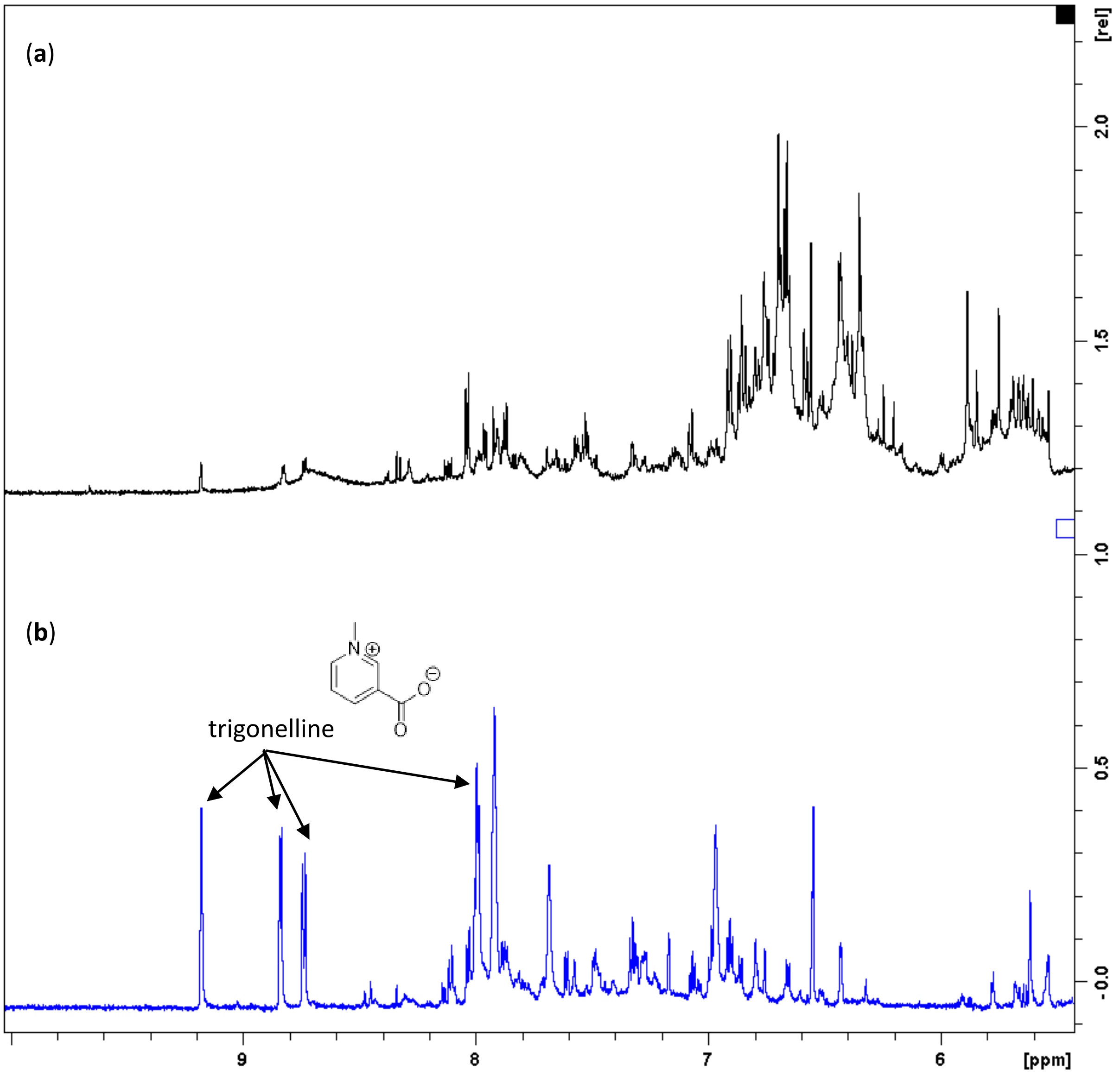

Supplementary Figure 4. ^{1}H NMR spectra of hull and cotyledon for lentil line CIPAL1422 grown at Ouyen showing downfield resonances associated with the major aromatic metabolite classes present: (**a**) spectrum of the lentil hull showing more phenolic resonances; and **(b)** spectrum of the lentil cotyledon showing predominance of the alkaloid trigonelline.

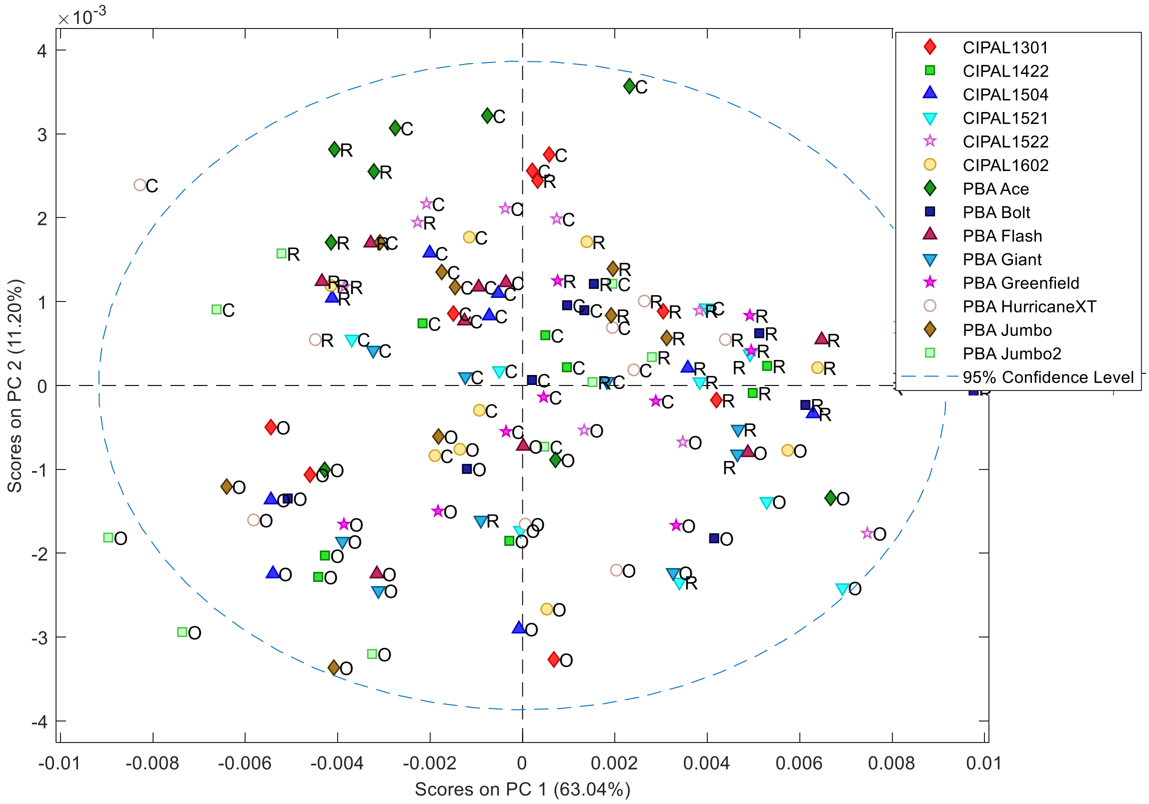

Supplementary Figure 5. PCA scores plot for cotyledon data coloured by genotype with site indicated by letter: O=Ouyen, C=Curyo and R=Rupanyup. Ouyen grown CIPAL 1522 lentils (+ve PC1) have more lipid and less carbohydrates than the PBA Jumbo2 (-ve PC1), refer to loadings plots in main text (Figure 3 b).

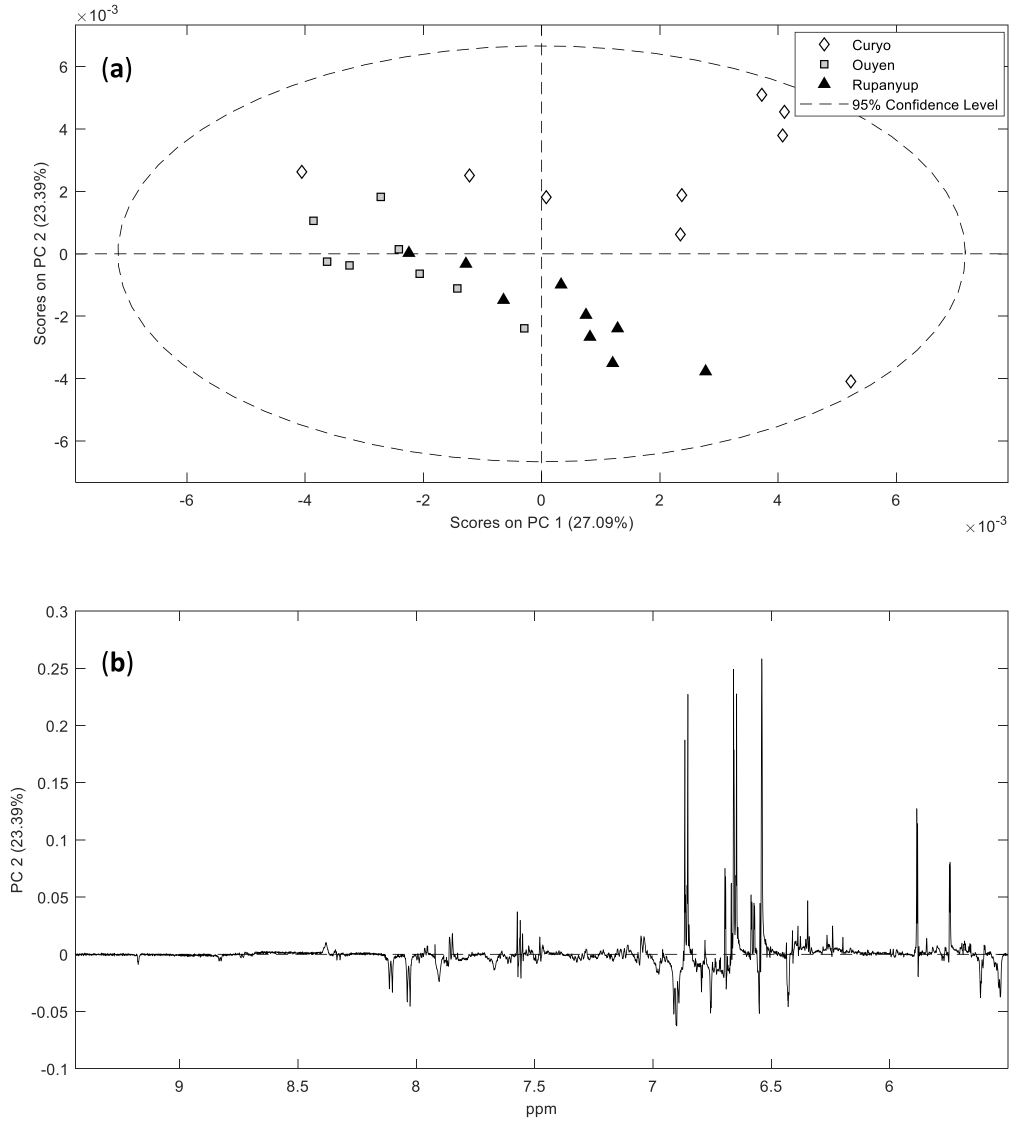

Supplementary Figure 6. PCA scores and loadings plot for the phenolic region of hull data for PBA Giant, PBA Flash and PBA Greenfield a: (**a**) scores plot showing Curyo separates from the other sites on PC2; and **(b)** loadings plot for PC2 demonstrating elevation in catechin/epicatechin derivatives.

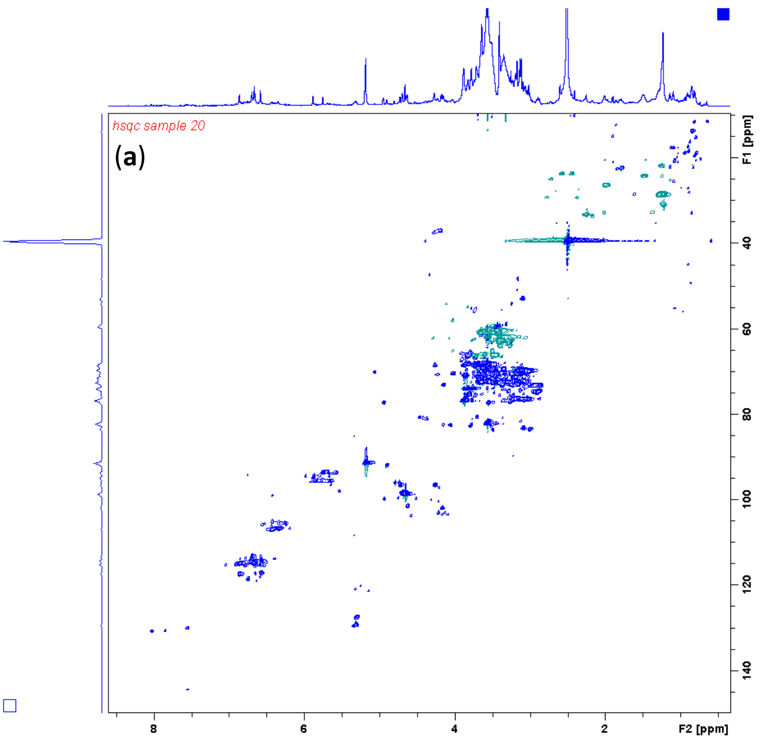

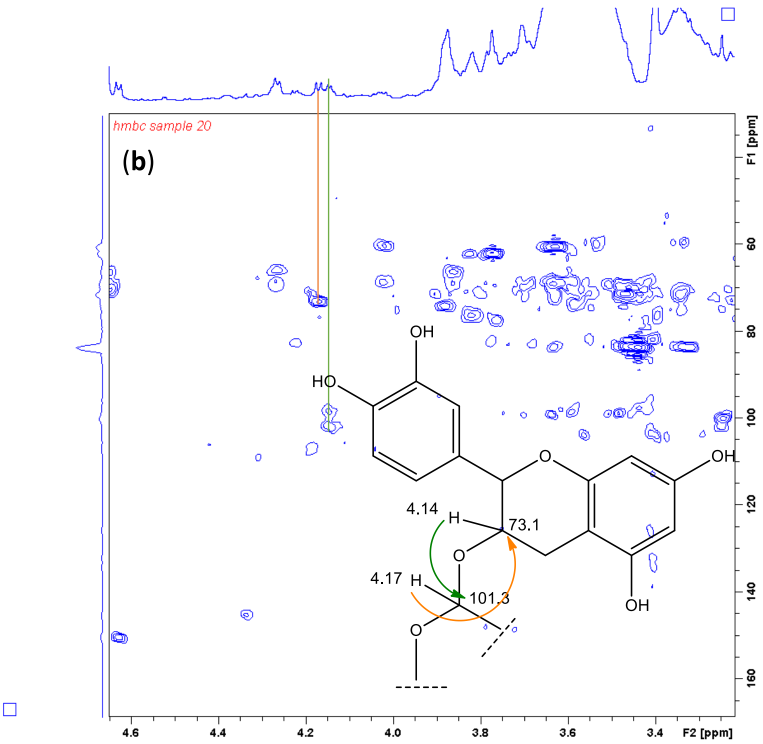

Supplementary Figure 7. 2D NMR data for a representative lentil sample: (a) gHSQC data for a hull sample. (b) gHMBC data for catechin/epicatechin highlighting correlation between an anomeric proton and the flavanol-3-ol carbon.

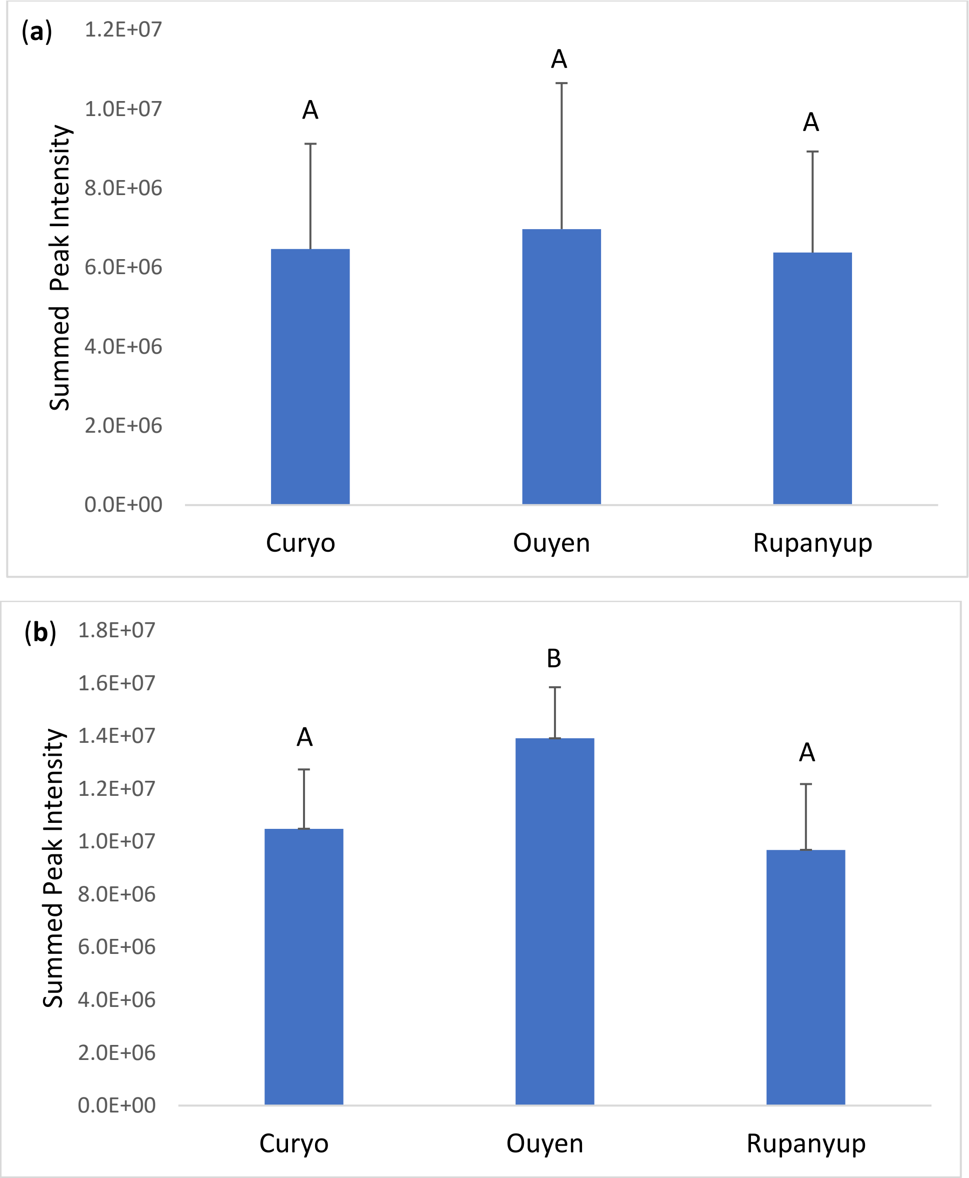

Supplementary Figure 8. Relative catechin content and standard deviation (error bars) in the lentil hulls by site: **(a)** all genotypes and all replicates, and **(b)** PBA Flash, PBA Giant and PBA Greenfield only. Different letters indicate significance at p=0.05 (Student's *t*-test).

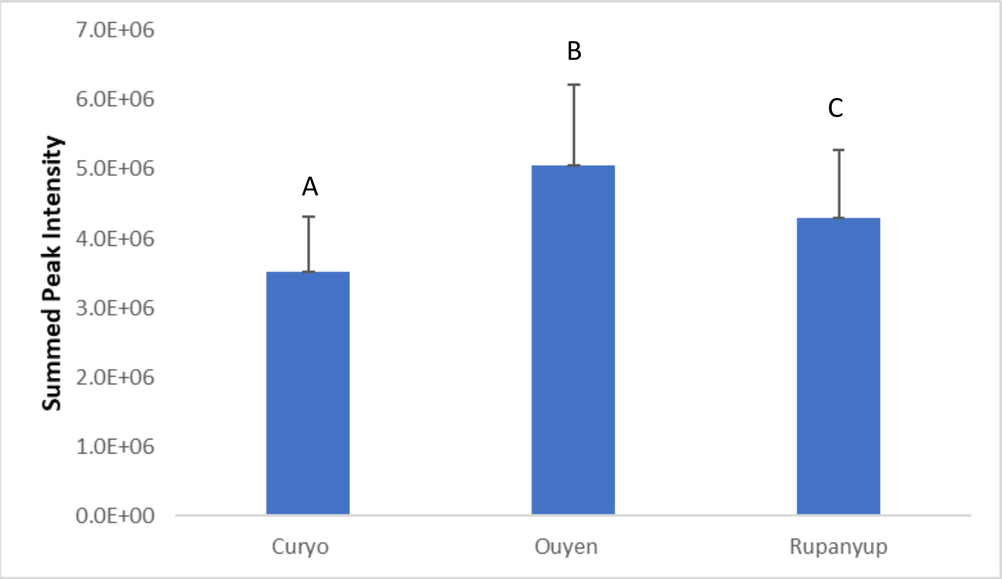

Supplementary Figure 9. Relative kaempferol content and standard deviation (error bars) in the lentil hulls by location (includes all samples). Different letters indicate significance at p=0.05 (Student's *t*-test).

Supplementary Tables 3 a-i. NMR data for standards, assigned by ^{1}H NMR (noesypr1d), 2D NMR (cosygpqf, hsqcedetgsisp2.2 and hmbcgpl2ndqf) and references where noted.

Supplementary Table 3a. NMR data for fructose (DMSO-d6, 700MHz)

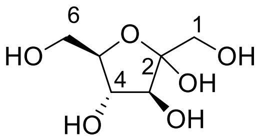

No	^{1}H δ (m, J *Hz*)	^{13}C (ppm, m)
C1	3.40 (br d, 11.)<br>3.27 (dd, 5.5, 11.0)	64.2
C2	-	98.0
C3	3.64	68.9
C4	3.57 (br m)	69.6
C5	3.57 (br m)	67.6
C6	3.78 (br d, 12.8)<br>3.42 (br d, 12.8)	62.8
C1-OH	4.48 (dd, 5.5)	-
C2-OH	5.11 (s)	-
C3-OH	4.20 (br d, 5.7)	-
C4-OH	4.35 (br d 5,2)	-
C6-OH	4.29 (br d 5.2)	-

Supplementary Table 3b. NMR data for α-glucose (DMSO-d6, 700MHz)

Ref: Gillet et al, Mag Res Chem. **1981** 17 (1) 28-36.

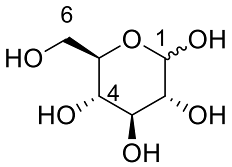

No	^{1}H δ (m, J *Hz*)	^{13}C (ppm, m)
C1	4.90 (br t, 4.2)	91.9
C2	3.10 (ddd, 6.7, 3.6, 0.6)	72.8
C3	3.41 (br m)	73.4
C4	3.03 (m)	70.9
C5	3.55 (br m)	72.4
C6	3.44 (br m)<br>3.59 (ddd, 11.5, 5.5, 2.3)	61.6
C1-OH	6.17 (dd, 4.2, 0.6)	-
C2-OH	4.40 (d, 6.7)	-
C3-OH	4.59 (br d, 4.7)	-
C4-OH	4.73 (br d 5.3)	-
C6-OH	4.32 (br t 5.8)	-

Supplementary Table 3c. NMR data for sucrose (DMSO-d6, 700MHz)

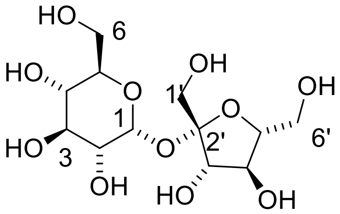

No	^{1}H δ (m, J *Hz*)	^{13}C (ppm, m)
C1	5.18 (br d, 3.3)	91.4
C2	3.18 (br dd, 9.6, 3.6)	71.4
C3	3.13 (br t, 9.1)	69.5
C4	3.65 (br d 7.6)	72.5
C5	3.47 (br m)	72.5
C6	3.50 (br m)<br>3.58 (br m)	60.2
C1’	3.41 (br s)<br>3.56 (br m)	61.8
C2’	-	102.5
C3’	3.88 (br d, 7.4)	76.7
C4’	3.78 (br t, 7.4)	74.0
C5’	3.57 (br m)	82.2
C6’	3.50 (br m)<br>3.58 (br m)	60.2

Supplementary Table 3d. NMR data for manitol (DMSO-d6, 700MHz)

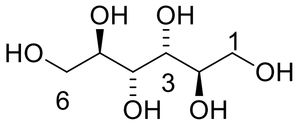

No	^{1}H δ (m, J *Hz*)	^{13}C (ppm, m)
C1, C6	3.38 (br m)<br>3.61(br m)	63.5
C2, C5	3.56 (br m)	71.1
C3, C4	3.54 (br t, 7.8)	69.5

Supplementary Table 3e. NMR data for inositol (DMSO-d6, 700MHz)

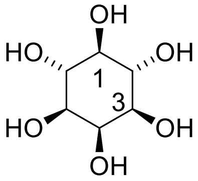

No	^{1}H δ (m, J *Hz*)	^{13}C (ppm, m)
C1	2.90 (t, 9.1)	74.9
C2, C6	3.34 (t, 9.1)	72.4
C3, C5	3.12 (br dd, 9.1, 2.5)	71.6
C4	3.70 (br s)	72.3

Supplementary Table 3f. NMR data for trehalose (DMSO-d6, 700MHz)

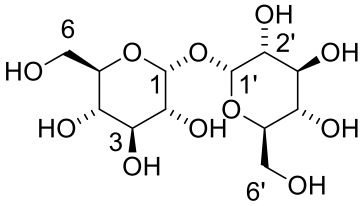

No	^{1}H δ (m, J *Hz*)	^{13}C (ppm, m)
C1/C1'	4.89 (d, 3.4)	93.5
C2/C2'	3.24 (dd, 9.5, 3.4)	71.1
C3/C3'	3.56 (t, 9.5)	73.3
C4/C4'	3.14 (br d, 7.6)	70.5
C5/C5'	3.65 (br m)	72.9
C6/C6'	3.48 (dd, 11.7, 4.9)<br>3.56 (br m)	61.2

Supplementary Table 3g. NMR data for raffinose (DMSO-d6, 700MHz)

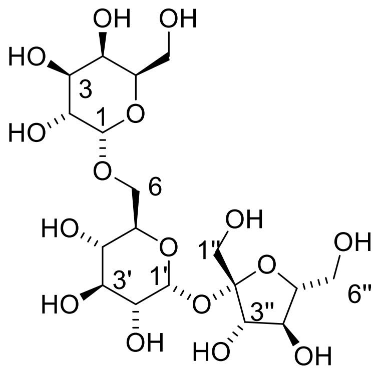

No	^{1}H δ (m, J *Hz*)	^{13}C (ppm, m)
C1	4.65 (br s)	98.8
C2	3.63 (br t, 6.2)	70.7
C3	3.83 (br t, 7.7)	74.0
C4	3.63 (br t, 6.2)	70.7
C5	3.47 (br m)	72.6
C1''	3.41 (br s)<br>3.57 (br m)	61.8
C1'	5.18 (br d, 3.5)	91.4
C2'	3.21 (br dd, 9.3, 3.5)	71.3
C3'	3.14 (br t, 9.3)	70.0
C4'	3.87 (br m)	71.0
C5'	3.47 (br m)	72.6
C6'	3.47 (br m)<br>3.71 (br m)	66.3
C1''	3.41 (br s)<br>3.56 (br m)	61.8
C2''	-	103.9
C3''	3.88 (br m)	76.8
C4''	3.83 (br t, 7.7)	74.0
C5''	3.58 (br m)	82.2
C6''	3.44 (br m)<br>3.50 (br m)	60.3

Supplementary Table 3h. NMR data for ciceritol (DMSO-d6, 700MHz)

Ref: Bernabé M et al, J. Agric. Food Chem. 1993, 42, 870-872

Complete assignment of ciceritol in our hands was not possible since the compounds was not pure and there were many overlapping resonances corresponding to oxymethines (67.0-77.7 ppm, 3.50 - 4.00).

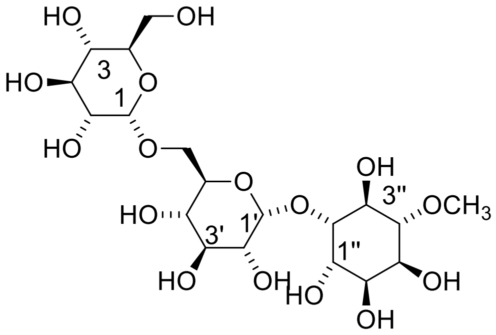

No	^{1}H δ (m, J *Hz*)	^{13}C (ppm, m)
C1	4.63 (br s)	98.8
C2	3.59 (m)	69.2
C6	3.41 (m)<br>3.60 (m)	61.9
C1’	5.18 (d,3.9)	91.3
C6’	3.50 (m)<br>3.61 (m)	66.0
C4’’	3.10 (br t, 9.4)	82.3
C5’’	3.54 (br m)	70.3
C4’’OCH_3	3.47 (s)	59.7

Supplementary Table 3i. NMR data for catechin (DMSO-d6, 700MHz)

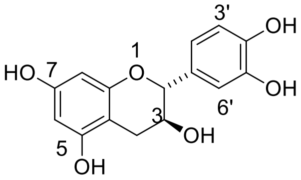

No	^{1}H δ (m, J *Hz*)	^{13}C (ppm, m)
C2	4.47 (d, 7.5)	80.7
C3	3.81 (br dd, 8.0, 5.4)	66.0
C4	2.35 (dd 16.0, 8.2)<br>2.65 (dd 16.0, 5.4)	27.6
C4a	-	99.0
C5	-	155.8
C6	5.88 (br d 1.9)	94.8
C7	-	156.5
C8	5.68 (br d 1.9)	93.5
C8a	-	155.2
C1’	-	130.7
C2’	6.59 (br dd, 8.0, 1.3)	118.1
C3’	6.70 (d, 8.0)	114.7
C4’	-	144.8
C5’	-	144.8
C6’	6.72 (br s)	114.2

Supplementary Table 3i. NMR data for epi-catechin (DMSO-d6, 700MHz)

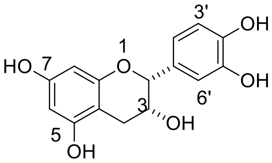

No	^{1}H δ (m, J *Hz*)	^{13}C (ppm, m)
C2	4.73 (br s)	77.7
C3	4.00 (br m)	64.6
C4	2.47 (dd 16.4, 3.4)<br>2.68 (dd 16.4, 4.5)	27.9
C4a	-	98.3
C5	-	155.5
C6	5.88 (br d 2.0)	94.8
C7	-	156.2
C8	5.71 (br d 2.0)	93.5
C8a	-	155.6
C1’	-	130.4
C2’	6.66 (m)	117.6
C3’	6.65 (m)	114.4
C4’	-	144.3
C5’	-	
C6’	6.89 (br s)	114.6
